# The 2016 HIGh Heels: Health effects And psychosexual BenefITS (HIGH HABITS) study: systematic review of reviews and additional primary studies

**DOI:** 10.1186/s12889-017-4573-4

**Published:** 2017-08-01

**Authors:** Max Barnish, Heather May Morgan, Jean Barnish

**Affiliations:** 1 Child Health, University of Aberdeen, Aberdeen, UK; 2 Health Services Research Unit, University of Aberdeen, Aberdeen, UK; 3 Centre for Gender Studies, University of Aberdeen, Aberdeen, UK; 4 Retired Health Visitor, London, UK

**Keywords:** Public health, High-heeled shoes, High heels, Social determinants of health, Freedom of choice, Osteoarthritis, Hallux valgus, Pain, Injury

## Abstract

**Background:**

High-heeled shoes (high heels) are frequently worn by many women and form an important part of female gender identity. Issues of explicit and implicit compulsion to wear high heels have been noted. Previous studies and reviews have provided evidence that high heels are detrimental to health. However, the evidence base remains fragmented and no review has covered both the epidemiological and biomechanical literature. In addition, no review has considered the psychosexual benefits that offer essential context in understanding the public health challenge of high heels.

**Methods:**

We searched seven major bibliographic databases up to November 2016, in addition to supplementary searches. We initially identified all review articles of any design that assessed either the psychosexual benefits or negative musculoskeletal health effects of high heels, the latter looking at both the epidemiological and biomechanical perspectives. We additionally considered additional primary studies on areas that had not been reviewed before or in which a marked lack of evidence had been noted. Data were extracted onto standardised forms. Proportionate second review was conducted.

**Results:**

A total of 506 unique records were identified, 27 full-text publications were screened and 20 publications (7 reviews and 13 additional studies) were included in our evidence synthesis. The most up-to-date epidemiological review provides clear evidence of an association between high heel wear and hallux valgus, musculoskeletal pain and first-party injury. The body of biomechanical reviews provides clear evidence of changes indicative of increased risk of these outcomes, as well as osteoarthritis, which is not yet evidenced by epidemiological studies. There were no reviews on psychosexual benefits, but all five identified original studies provided evidence of increased attractiveness and/or an impact on men’s behaviour associated with high heel wear. With regard to second-party injury, evidence is limited to one descriptive study and eight case reports.

**Conclusions:**

Our evidence synthesis clearly shows that high heels bring psychosexual benefits to women but are detrimental to their health. In light of this dilemma, it is important that women’s freedom of choice is respected and that any remaining issues of explicit or implicit compulsion are addressed.

## Background

### Cultural and policy context

High-heeled shoes (high heels) are a form of footwear that raises the heels of the wearer’s foot substantially above the level of the toes, making the wearer taller. They have a long history in human civilisation [1]. An earlier form of high heels were worn by men in the medieval period, when their function was largely practical, that is to say to raise the wearer above the filth of medieval streets [2]. In modern society, high heels have become a fashionable symbol of modern (heteronormative) female sexuality [3], and are regularly worn by a considerable proportion of women [4]. Fashion operates by capitalizing on concepts of social compliance and conformity [5, 6], potentially mediated through celebrity influence [7, 8] and the expectation to perform normalised gender roles [9, 10]. Many feminist theorists have argued that gendered violence occurs when women become disabled by their clothing and footwear [11], when standards of beauty define dimensions of physical freedom [12, 13].

Although many women like wearing high heels for various reasons, this cultural context raises the possibility of some women wearing high heels when they have not chosen freely to do so. One online opinion poll estimates that the proportion of women whose high heel wear is mainly due to social expectation rather than their own free choice may be around a third [14]. This would represent a substantial minority, and is around the same as the proportion this poll reports have ever been required to wear high heels at work.

Moreover, political features and approaches have been shown to be an important determinant of population-level health outcomes [15]. One way in which such influences may operate is through the extent to which a government decides to intervene in societal matters. One major policy controversy in some localities recently has been whether employers are legally allowed to, or indeed should be allowed to, stipulate that female staff wear high heels to work. In the United Kingdom, the Government has published a response [16] stating that it will work to develop guidelines and raise awareness of this issue, as others have also done, to ensure women are not discriminated against in the workplace. Nevertheless, there has been some media confusion about the details of this response and some commentators have criticised the decision to rely on the Equality Act (2010) rather than propose new legislation. In comparison, the Canadian province of British Columbia has chosen to make an amendment to existing legislation to specifically prohibit employers from requiring female staff to wear high heels [17]. Current policy discussions have largely not considered issues relating to entertainment venue or licensed social event admission policies, although the policy of the Cannes Film Festival has been very controversial [18].

### Existing evidence

The popular view that wearing high heels increases women’s attractiveness to men is supported by scientific evidence [19, 20]. Although this is not a health outcome, it is socially relevant and may be of interest to the public health community since it is the tension between psychosexual benefits and negative musculoskeletal (MSK) health effects that makes high heels a topic of substantial public health interest and also of interest with regard to the study of the social determinants of health outcomes. A large number of studies have been conducted to examine the relationship between high heels and MSK health. There are two dominant approaches as discussed before [21] each with their associated strengths and limitations: the experimental biomechanical approach and the epidemiological approach. For example, the former approach provides clearer insight into causality, while the latter does not rely on proxy markers of clinical outcomes.

Thorough review articles have been published from both perspectives. The most recent biomechanical review [22] found evidence of qualitatively consistent alterations to the neuromechanics of walking gait and the kinetics and kinematics of bodily structures from the toes to the spine in ways that could be seen as biomechanical markers of MSK conditions such as hallux valgus (HV) and osteoarthritis (OA). This was a narrative non-systematic review published in 2014 with the most recent included study being published in 2012. Meanwhile, the most recent epidemiological review found evidence of an association between high heel wear and MSK pain, HV and first-party injury. Nevertheless, associations with OA and second-party injury were inconclusive. This was a narrative systematic review published in 2016 with a final search date of July 2015.

### Gaps in knowledge and the aims of our systematic review

The evidence base has remained fragmented as no prior review article has integrated both the epidemiological and biomechanical perspectives. Moreover, no prior review has considered the psychosexual benefits, which provide important social context to this important area of public health debate. Conducting an integrated review of these perspectives has been identified [21] as a research priority. Indeed, we were aware of a number of prior reviews on different aspects of this topic that we could synthesize. Therefore, we present the first systematic review of reviews on high heels. Our review is the first to consider both the epidemiological and biomechanical perspectives on the negative MSK health effects of high heels. In addition, our review is the first to present evidence on the psychosexual benefits associated with high heel wear, thereby providing essential context in understanding the public health challenge of high heels. To further strengthen our evidence synthesis, we added additional primary studies on areas in which no review had been conducted or a marked lack of prior research had been identified.

## Methods

### Design

The 2016 HIGh Heels: Health effects And psychosexual BenefITS (HIGH HABITS) study used a systematic review approach following PRISMA [23] as appropriate. In light of the existence of a number of prior reviews on aspects of this topic, albeit a stark lack of integration of different aspects, our systematic review initially sought to synthesise evidence from available reviews. Subsequently, additional primary evidence was added on areas identified to be lacking in research and/or prior reviews. The search strategy was designed by MSB (lead reviewer) and JB, who conducted proportionate quality control on review procedures. Any discrepancies were resolved by discussion.

### Data sources

MEDLINE (Ovid), EMBASE (Ovid), AMED (Ovid), PsycINFO (Ovid), Cochrane Central (Ovid), CINAHL (EBSCO) and Web of Science (Thomson Reuters, excluding patents) were searched up to November 2016. As per Barnish and Barnish [21], we applied the following keywords to all databases: “positive heel inclination”, “high heel”, “high-heeled”, ‘“high heeled”, “wedge heel”, “platform heel”, “platform shoe”, “stiletto” and “elevator shoe”. The only change we made was to add quotation marks to increase search specificity. Supplementary searches were conducted in Google Scholar (GS), Directory of Open Access Journals (DOAJ) and bibliographies of relevant articles.

### Inclusion criteria

We included review studies of any design (non-systematic review, systematic review or meta-analysis) that were:

- Published as full articles in English, French, German, Spanish, Italian, Dutch or Portuguese language peer-reviewed scientific journals
- Provided evidence to associate high heel wear (not revised high heels or orthotics) with at least one of psychosexual benefits, or osteoarthritis (OA), hallux valgus (HV), musculoskeletal (MSK) pain or first- or second-party injury from either an epidemiological or biomechanical perspective. Indirect injuries such as road traffic accidents were not considered, as high heels and driving safety is a topic that would require a review in its own right
- Assessed human participants without prior history of clinically significant MSK conditions or other serious medical conditions likely to affect outcomes

We also considered individual primary studies that:

- Addressed an aspect on which no review had been published (which turned out to be the psychosexual benefits), or addressed second-party injury, which had been noted [21] to be an area in which research was particularly lacking
- Used a quantitative design, with the exception of second-party injury, for which case reports were also considered due to a prior finding [21] of almost no prior population-based studies

### Data extraction

The following data were extracted for review articles:

- Bibliographic details
- Language of publication
- Review design
- Search date (or if not available, year of last included publication)
- Inclusion criteria
- Principal findings

The following data were extracted for primary studies:

- Bibliographic details
- Language of publication
- Country of study
- Study design
- Participants
- Exposure measurement
- Outcome measurement
- Principal findings

Heel height measurements expressed in inches were converted to cm at 1 in. = 2.54 cm. Risk of bias assessment could not be conducted in a way that would offer useful comparability due to a wide range of different designs.

## Results

### Search results

Database searches yielded 923 records (MEDLINE 223, EMBASE 251, AMED 54, PsycINFO 30, Cochrane Central 12, CINAHL 2 and Web of Science 351), which yielded 492 unique records following deduplication. Supplementary searches yielded an additional 14 records (3% of unique records), providing a grand total of 506 unique records. Ten reviews proceeded to full-text screening and seven met the inclusion criteria for our review – of these, two [21, 24] were systematic reviews without meta-analysis and five [22, 24–28] were non-systematic reviews. There were no meta-analyses. All seven reviews were published in English, although one [28] was also available in Portuguese. Ninety-six unique publications were reviewed across these seven review articles.

**Fig. 1:**
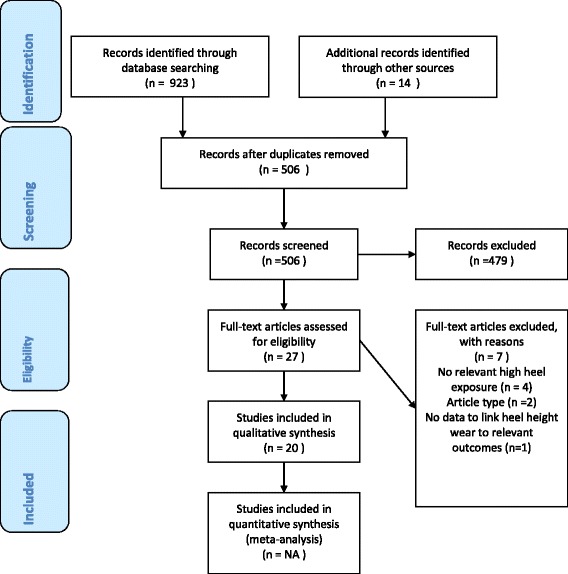
PRISMA 2009 Flow Diagram

Seventeen additional primary publications proceeded to full-text screening and 13 met the inclusion criteria for our review, all published in English. Eight publications [29–36] were case reports of second-party injury reporting a total of 10 cases from three countries (UK, Denmark and Ireland). Five publications [19, 20, 37–39] reported psychosexual studies from three countries (France, UK and Egypt). There were no further non-case report studies on second-party injury. All reports were considered separate studies since different analyses were reported and our approach did not involve meta-analysis. The article selection process is shown in Fig.1, study results in Table 1, study characteristics in Table 2 and studies excluded at full-text review with reasons in Table 3.

**Table 1:** Results of included studies

Part A. Results of included reviews	
Study	Results
Barnish and Barnish^a^, 2016 [21]	Evidence from 18 studies was included (total sample size 14,647). Six studies (33%) were assessed as high quality, eight (44%) as moderate quality and four (22%) as low quality. Studies varied in terms of their populations, ranging from young women to the elderly, while others were whole-population studies including children. Three out of four studies found that high heels were associated with HV. Zero out of two studies found an association with OA. Three out of five studies found an association with MSK pain and this evidence was strengthened when study quality was taken into consideration. Seven out of eight studies found an association with first-party injury – two of these studies profiled emergency department presentations and found a considerable proportion of children among presenting cases. Only one study provided data on second-party injury and the reported injury toll was low. Generally, this review provides good epidemiological support for the idea that wearing high heels increases a woman’s risk of suffering MSK ill-health.
Cowley et al., 2009 [25]	Evidence from 38 publications is included. The body of evidence supports the view that high heels are associated with pain, HV and ankle inversion injuries. The majority of studies found alterations in foot and ankle kinematics, kinetics, knee and hip flexion, gait, posture and balance. Evidence for spinal alterations was less conclusive.
Cronin, 2014 [22]	Evidence from 43 publications is included. The body of evidence supports the view that wearing high heels leads to qualitatively consistent alterations to the neuromechanics of walking gait and the kinetics and kinematics of bodily structures from the toes to the spine in ways that may be seen as biomechanical markers of MSK conditions such as HV and OA.
Murley et al.^a^, 2009 [24]	Evidence from four studies on the effect of heel height was included. Most studies were on young adults. Generally, there was good evidence of alterations in lower limb and low back muscle activity, although one study found no significant difference.
Riskowski et al., 2011 [26]	Evidence from three studies on the effect of heel height is included. The single study on OA did not found an association with high heel wear. One of two studies on pain found an association.
Russell, 2010 [27]	Evidence from nine studies (one only available as a conference abstract) was included (total female sample size 182). Increased lumbar lordosis (potentially accompanied by increased pelvic tilt) would be expected to indicate increased risk of low back pain. This result was found in two out of nine studies, with a trend to an effect in younger female participants in one further study. The author argues for a disconnect between scientific evidence and Internet content/clinician opinion regarding the potential role of high heels in low back pain.
Silva et al., 2013 [28]	This could be considered a ‘semi-systematic’ review. Evidence from 20 studies fulfilling inclusion criteria, and 5 other studies, not fulfilling the criteria but considered useful, was included. This review focused on adolescents (aged 10 to 19). There was evidence that high heel wear in adolescents can lead to postural disorders affecting head positioning, the back, pelvis and knee. Heel height and width were identified as key factors in the emergency of postural changes and body imbalance.
Part B. Results for primary studies on psychosexual benefits associated with wearing high heels	
Guéguen, 2015 [20]	Male participants were more likely to answer a survey on gender equality when the female confederate was wearing high heels than flat shoes (83% vs 47%). The difference between high and medium (63%) heels approached significance, but the difference between medium and flat was not significant. Male participants were more likely than female participants to answer a survey on food consumption habits presented by a female confederate. There was a significant effect of shoe condition for men (flat = 42%, medium = 60% and high heel = 82%), but not women (flat = 32%, medium = 37%, high = 30%). Male participants were more likely than female participants to help when a female confederate dropped a glove – there was a significant effect of shoe condition for men (flat = 62%, medium = 78% and high heel = 93%) but not for women (flat = 43%, medium = 50% and high 52%). Male participants were significantly more likely to approach a female confederate in a bar if she was wearing high heels than either medium (*p* = 0.02) or flat (*p* < 0.001) shoes – there was no significant difference between flat and medium (*p* = 0.26).
Guéguen and Stefan, 2015 [37]	Male participants were more likely to smile back at a female confederate if she was wearing high heels (56%) than medium heels (36%, *p* = 0.04) or flat shoes (30%, *p* < 0.01) – there was no significant difference between flat and medium (*p* = 0.52). Male participants were more likely than female participants to answer a survey on gender equality presented by a female confederate – there was a significant effect of shoe condition for men (flat = 48%, medium = 60%, high heel = 82%) but not women (flat = 42%, medium = 38%, high = 46%). Male participants’ judgements of a photograph of a young woman presented on a computer screen were influenced with regard to sexiness (*p* = 0.004), bust attractiveness (*p* < 0.001), overall attractiveness (*p* < 0.001), beauty (*p* = 0.01), desire to meet (*p* = 0.007) and attractiveness to other men (*p* < 0.01) depending on whether she was wearing flat shoes or high heels, even though the shoes were not shown in the photograph. There was no statistically significant effect on buttock attractiveness, probability of being in a couple, academic level, probability of having a child, expected dating acceptance, youth or age estimation.
Guéguen et al., 2014 [38]	Male participants’ judgements of a photograph of a young women presented on paper were influenced with regard to sexiness (*p* < 0.001), prettier legs (*p* < 0.001), prettier buttocks (*p* < 0.001), more elegance (*p* < 0.001), more good-looking (*p* < 0.001), youth (*p* < 0.001), being selected by others (*p* < 0.001) and being selected for an album (*p* < 0.001) depending on whether she was wearing flat shoes or high heels, even though the shoes were not shown in the photograph. These effects were all also found at *p* < 0.001 for female participants’ ratings.
Maarouf, 2015 [38]	When asked whether wearing high heels increased their attractiveness, 64% of surveyed businesswomen said yes, 27% said maybe and 9% said no. Among ‘worker’ women, 42% said yes, 45% said maybe and 13% said no. Among female university students, 66% said yes, 24% said maybe and 10% said no.
Morris et al., 2013 [19]	When assessing point-light displays of females walking, attractiveness index scores were higher in the high heel condition than the flat condition (*p* < 0.001). Female raters assessed the female walkers as being more attractive than male raters (*p* < 0.001). There was no statistically significant interaction between rater gender and shoe condition. Male and female raters were more accurate in correctly identifying female walkers as female when they were wearing high heels (*p* < 0.01). In this second experiment, raters were told there would also be male walkers, although in reality there were no males.
Part C. Description of case reports on second-party injury	
Study	Case description
Ahmed, 1964 [29]	**Case 1**: 31 year old man struck on the head with a stiletto heel after a fracas in a public house (a traditional type of bar in the UK). Radiograph revealed a small stellate depressed fracture to left parietal area with a small scalp laceration. Epileptic and dysphasic symptoms encountered. Patient recovered from operation and displayed no neurological abnormality at outpatient follow-up in February 1964, although abnormal EEG activity persisted in the left temporo-parietal area. **Case 2**: 36 year old woman struck on the head by another women’s stiletto heel following a street fight. She suffered aphasia and right hemiplegia, with radiographs showing a depressed fracture in left parietal parasagittal area. Following operation, a gradual full recovery was experienced.
Cleary et al., 2006 [30]	**Case:** 16 year old female stepped on by stiletto heel of a fellow dancer on a dance floor. Penetration of right orbit, presented with headache and right eye pain. Seven months later, although visual acuity had improved, elevation and abduction were limited, with persisting ptosis. Surgery was planned to correct ocular position.
Engelhart and Bliddal, 1997 [31]	**Case 1:** 53 year old woman stepped on by another women’s high heels. Presented to doctor with oedema and pain, no fracture found on x-ray. Wound gradually healed after 4 months and pain slowly regressed. **Case 2:** 50 year old women stepped on by another women’s high heels. Presented to doctor with oedema and pain, no fracture found on x-ray but infection suspected. Patient generally healthy at 6 month follow-up.
Fry, 1959 [32]	**Case**: 14 year old girl stepped on by another girl’s stiletto heel in a bus. Marked tenderness and swelling to the foot developed 2 days later and the girl could not use her foot. Penicillin was prescribed when no healing had taken place 10 days after. After five more days, improvement was noticed, but over 5 weeks after the injury, the girl was still having great difficulty walking. The clinician considered the girl fortunate not to suffer severed tendons.
Jewsbury and Haslett, 2011 [33]	**Case**: 43 year old woman stamped on the head with a stiletto heel. Presented appearing intoxicated but denied drinking or illicit drug use. After discharge, re-presented a month later with diplopia and headaches, mild dysphasia and irritability and ocular motility consistent with acquired Brown’s syndrome. Scans showed comminuted fracture of left medial orbital roof and cerebral laceration of left frontal lobe.
Joyce and O’Shaughnessy, 2016 [34]	**Case**: 22 year old woman stood on by another women’s stiletto heel at a bar. Swelling immediately encountered but minimal pain. On presentation, the hallux was grossly swollen with distal necrosis of the nailbed and pulp, accompanied by cellulitis. Skin graft operation was performed. Full healing was encountered.
Missen, 1964 [35]	**Case**: 54 year old man struck on the head with a stiletto heel by a female relative in a domestic fracas. Patient fell unconscious but was conscious on presentation to hospital. No neurological abnormalities were encountered. There was a scalp laceration in the right posterior parietal region. X-ray showed a deeply depressed bone fragment. An operation revealed a skull defect about 0.75 cm in diameter. The patient had recovered by discharge date and no further symptoms presented during a 1.5 year follow-up period.
Stables et al., 2005 [36]	**Case**: 23 year old man struck on side of head with a stiletto heel. Initially presented with a small wound and was discharged. He became increasingly unwell and encountered speech difficulties. Scans revealed minor depressed skull fracture of left parietal bone and underlying contusion of the parietal lobe. Upon arrival at neurosurgical unit, he presented with mild dysphasia which worsened. Symptoms presented consistent with cerebritis. Following craniectomy, he improved and the dysphasia had resolved at 12-month follow-up.

^a^systematic review, *HV* hallux valgus, *MSK* musculoskeletal, *OA* osteoarthritis

*EEG* electroencephalogram

**Table 2:** Characteristics of included studies

Part A – review articles						
Authors	Language of publication		Review design	Search date/year of last included publication	Inclusion criteria^b^	
Barnish and Barnish, 2016 [21]	English		Systematic review	July 2015	Full-text original research studies published in peer-reviewed journals; published in English, French, German, Spanish, Italian, Dutch or Portuguese; able to be retrieved; providing data to associate high heel wear with at least one of osteoarthritis, musculoskeletal pain or hallux valgus verified by clinical diagnosis or assessment, or first- or second-party injury; assessing human participants without prior history of musculoskeletal conditions or other serious conditions likely to affect outcomes; using any quantitative epidemiological design	
Cowley et al., 2009 [25]	English		Non-systematic review	2007^a^	Not stated: the research question was about the effect of high heels on female gait and posture	
Cronin, 2014 [22]	English		Non-systematic review	2012^a^	Not stated: the research question was about how high heels affect female gait	
Murley et al., 2009 [24]	English		Systematic review	2007	Main outcome for muscle activity was EMG or muscle activity during walking or running; assessed changes in foot posture, orthoses or footwear; statistical testing was conducted; human participants without neurological disease; not a single case report or ‘n of 1 study’	
Riskowski et al., 2011 [26]	English		Non-systematic review	2010^a^	Not stated: This is a wide-ranging review that covers both problematic effects of footwear on health and how footwear can be used as therapy	
Russell, 2010 [27]	English		Non-systematic review	June 2010	English-language publications about the relationship between high heels and lumbar lordosis	
Silva et al., 2013 [28]	English (also available in Portuguese)		Non-systematic review	2011	Articles published between 1980 and 2011, regardless of study design with participants partly or entirely females aged between 10 and 19; assessing posture of spine and lower limbs, location of centre of gravity and effects of high heels on the adolescent musculoskeletal system. It is noted that 5 studies were added that did not fulfil the criteria	
Part B – primary studies						
Authors	Language of publication	Country	Study design	Participants	Exposures	Outcomes
Guéguen, 2015 [20]	English	France	Psychology experiment	Random selection. Experiment 1: men aged 25–50. Experiment 2: men and women aged 25–50. Experiment 3: men and women aged 20–45. Experiment 4: men aged 20–28. Between one and four female confederates were used, mean age 19, height without heels 167–168 cm, weight 54-57 kg.	Flat shoe vs medium (5 cm) heel vs high (9 cm) heel	Participation in surveys, helping the confederate when she has dropped a glove and approaching her in a bar
Guéguen and Stefan, 2015 [37]	English	France	Psychology experiment	Random selection. Experiment 1: men aged 18–35. Experiment 2: men and women aged 25–50. Experiment 3: male undergraduate students, mean age 20. Experiments 1 and 2: between one and four female confederates were used, mean age 19, height without heels 167-169 cm, weight 55-58 kg. Experiment 3: one 30 year old women was used as the target.	Experiments 1 and 2: Flat shoe vs medium (5 cm) heel vs high (9 cm) heel. Experiment 3: Flat shoe vs high (9 cm) heel	Smiling back, participation in a survey and attractiveness ratings
Guéguen et al., 2014 [38]	English	France	Psychology experiment	Male and female undergraduate business students aged 18–22. One 20 year old woman was used as the target.	Flat shoe vs high (9 cm) heel	Attractiveness ratings
Maarouf, 2015 [39]	English	Egypt	Cross-sectional	3 groups: businesswomen, female ‘workers’ and female university students	Heel height: high or not (no explicit cut-off)	Self-rated attractiveness, by questionnaire
Morris, 2013 [19]	English	UK	Psychology experiment	No specific inclusion criteria for raters. Walkers were an opportunity sample of young women who wore high heels at least weekly	Flat shoe vs high heel (6 cm)	Attractiveness index; correct gender identification

Characteristics could not be tabulated in this way for case reports

^a^ last included publication (used where no search date is stated); ^b^ inclusion criteria for the whole review as published – it may be part of this review that is used for the current review of reviews; *EMG* Electromyography

**Table 3:** List of publications excluded at full-text screening with reasons

Authors	Title	Journal	Bibliographic details	Reason for exclusion
Reviews				
Hannan MT	Epidemiologic perspectives on women and arthritis: an overview	Arthritis Care Res (Hoboken)	1996; 9: 424–34	Does not discuss high heels
Macfarlane GJ	The epidemiology of chronic pain	Pain	2016; 157: 2158–9.	Does not discuss high heels
Menz HB	Chronic foot pain in older people	Maturitas	2016; 91:110–4.	Does not reference any primary studies on high heels
Additional primary studies				
Domjanić J, Ujević D, Wallner B, et al.	Increasing women’s attractiveness: high heels, pains and evolution – a GMM based study	(NA)	Book of Proceedings of the 8th International Textile, Clothing and Design Conference, 2016.	Not peer-reviewed journal article
Ferrie EP, Erskine I	Stilettoed in the sixties	Emerg Med J	2006; 23: 240–1	Commentary on prior case reports, not new case report
Itshayek E, Gomori JM, Spektor S, et al.	Stiletto stabbing: penetrating injury to the hypothalamus with hyperacute diabetes insipidus	Clin Neurol Neurosurg	2010; 112: 924–6	Stiletto knife not shoe
Voracek M, Fisher ML, Rupp B, et al.	Sex differences in relative foot length and perceived attractiveness of female feet: relationships among anthropometry, physique and preference ratings	Percept Mot Skills	2007; 104: 1123–38	No data to associate high heel wear with attractiveness outcomes

### Reviews

Two reviews provided epidemiological data. Riskowski et al. [26] assessing literature up to 2010, did not focus primarily on high heels and only included three studies on heel height, reaching inconclusive conclusions regarding pain and OA. Meanwhile, a systematic review by Barnish and Barnish [21] up to July 2015 demonstrates how the field has progressed to provide clear evidence that high heels are associated with MSK pain, HV and first-party injury, including to children. There remained no clear epidemiological evidence of a link with OA and second-party injury. Only one descriptive study [40] had presented second-party injury data, the toll was relatively low and these were excluded from further analysis in the paper by Williams and Haines.

The remaining reviews focused on biomechanical aspects. Generally, across reviews, there was good evidence of considerable kinetic and kinematic alterations associated with high heel wear that can be considered markers of increased first-party injury risk and MSK conditions such as OA, HV and MSK pain. Silva et al. [28] provide valuable insights into postural disorders related to high-heel wear among adolescent girls (aged 10–19). Increased heel height and decreased heel width were identified as key predictors of negative outcomes. Looking specifically at lumbar lordosis, considering literature up to 2010, Russell [27] found inconclusive results. Meanwhile, Cronin [22] found qualitatively consistent alterations in kinetics and kinematics from the spine to the toes, although some studies were not in agreement on lumbar lordosis. More detail on the findings of each review can be found in Table 1 Part A.

### Additional primary studies

There were no prior reviews about the psychosexual benefits associated with high heel wear. Five studies addressed this aspect. All five studies presented evidence of such psychosexual benefits. One study from Egypt [39] assessed women’s own views as to whether wearing high heels increased their attractiveness, finding this effect among businesswomen, female employees and female university students alike. The remaining studies assessed judgements of attractiveness made by others or influence upon the behaviour of others. High heels were generally found to have a much greater effect than medium heels. A series of studies from France [20, 37, 38] found that attractiveness ratings, made by both male and female participants with regard to a photograph of a woman, were influenced by high-heel-associated postural changes, even though her shoes were not visible, whereas whether or not a man was willing to help a woman was only influenced by the confederate’s (a person who participates in a psychological experiment pretending to be a participant but is working for the researcher) heel height in male participants. Morris et al. [19] also noted that raters were more likely to misclassify a female walking in light-based silhouette form in a point-light task as male if she was wearing flat shoes as opposed to high heels. More details on the findings of the psychosexual studies can be found in Table 1 Part B.

The only eligible additional studies identified on second-party injury were case reports [29–36]. Eight case reports were included, presenting 10 cases. Injuries requiring emergency department attention were sustained of both deliberate and accidental etiology. Accidental causes were being stepped on by a stiletto heel in the eye after falling on a dancefloor, two unspecified cases of being stepped on by stiletto heels, a young girl suffering foot injury requiring emergency department attention after being stepped on by a stiletto heel when someone else was getting off the bus, and a young woman being stepped on by another woman’s stiletto heel at a bar. More detail on each case can be found in Table 1 Part C.

## Discussion

### Summary of findings

Here we present the first systematic review of reviews about high heels. We address both the biomechanical and epidemiological perspectives and offer the first review of the psychosexual literature, which provides essential context in which to situate the findings on negative health effects, and understand the public health issues and dilemmas they pose for society. The biomechanical literature is now clear that wearing high heels causes substantial kinetic and kinematic alterations in the MSK system, ranging from the spine to the toes. These alterations increase the risk of MSK conditions such as OA, HV and MSK pain as well as first-party injury. The epidemiological literature also now clearly says that wearing high heels is associated with increased risk of HV, MSK pain and first-party injury, although the risk of first-party injury requiring emergency department attention is at most moderate. However, there is still no clear epidemiological evidence of an association between high heel wear and OA. Therefore, the suggestion from a large volume of biomechanical evidence dating back to the seminal paper in the Lancet in 1998 by Kerrigan et al. [41], that high heel wear may predispose women to OA has not been confirmed in population-based studies. Evidence from one observational study [40] and a series of case reports [29–36] shows that high heels, and in particular stilettos, can cause second-party injuries requiring emergency department attention. However, current evidence does not allow a robust quantification of the magnitude of risk.

### Methodological considerations of the topic

The included literature benefits from a large number of reviews, although only two were systematic reviews. Compared to non-systematic reviews, systematic reviews have a more reproducible methodology and are less prone to selection bias [42]. The strengths and limitations of the epidemiological and biomechanical approaches have been discussed before [21]. High heel wear is a very common exposure [4] by epidemiological standards, being more common than very common health conditions such as childhood asthma or other negative health-related behaviours such as smoking [43]. It is challenging to find a truly non-exposed group. Moreover, studies have tended to focus on relative rather than absolute risk measures. Moreover, two injury studies [40, 44] included in the Barnish and Barnish review [21] used emergency department databases which may be subject to misclassification and/or underreporting bias and only provide insight into the extreme end of the injury spectrum. Meanwhile, psychosexual findings are limited by a failure to consider platforms and heel shape as well as an upper heel height bound of 9 cm. No study assessed the role of the respective heights of men and women. This could be relevant since there is a preference in heterosexual partnerships for the male to be taller than the female [45, 46], and very high heels may eliminate this difference since young women in Australia, the United States and England [47–49] are on average less than 15 cm shorter than young men. Indeed, research into this area has a clear heteronormative bias and does not consider alternative sexualities.

### Social context

High heels are a challenging topic due to the tension between health and psychosexual considerations. The evidence synthesised in our review shows that wearing high heels increases women’s attractiveness to men and can reward female wearers with other psychosexual benefits in terms of male attention and their own view of their beauty. This poses a potential opportunity or a dilemma, which many women face regardless of their sexuality. We hope our review can provide a useful resource for clinicians such as podiatrists and MSK specialists in discussing in gender sensitive ways high-heel related conditions and injuries with which their clients present. It is important that women’s footwear choices are respected and that they are not pressurised into wearing high heels against their will. Unstated social expectation and pressure, mediated by social compliance [5] and celebrity influence [7, 8] is clearly a complex challenge that is difficult to confront. Continuing to raise awareness of the health issues associated with high heels appears the most useful solution to seeking to maximise freedom of choice. Important policy developments have occurred recently in different localities [16, 17], although challenges remain regarding compliance with legislation and also compulsion that occurs beyond the workplace, which may be covered by equality legislation that refers to provision of goods and services.

High heels are currently one of the few ‘adult’ items allowed to be sold to children in countries such as the UK, and as Silva et al. [28] show us, this can lead to substantial health issues. Adolescents are particularly prone to peer pressure [50] and there is evidence that health may play a limited role in female teenagers’ shoe choices in particular [51]. Indeed, increased adolescent high heel wear has been observed in recent years [52]. It has been suggested [53] that education on their health effects, for example as part of Personal and Social Education, could be useful. Moreover, it may be worthwhile considering whether it is appropriate that high heels can be sold to and for the wear of people who are still skeletally immature.

### Future research directions

Further research is required into OA risk from an epidemiological perspective, second-party injury (perceptions and observations, related to high heels and potentially other glamorous women’s outfit types) and the competing influences on women’s freedom of choice in shoe selection, potentially paralleling Elliott-Green et al. [54]. It is also important, either from academic studies, or by monitoring the media, to assess as time progresses how issues of compulsion (explicit or implicit) are addressed. Moreover, academics should more frequently take the potential mediating effects of high heels into account when studying or reviewing the MSK health of female participants.

### Strengths and limitations of our review

The key scientific strength of our work is that it brings together for the first time three hitherto disparate bodies of evidence – the epidemiological, biomechanical and psychosexual – on a topic of current social interest. Moreover, we believe our work is of methodological value and offers an evidence synthesis approach that we hope will be of interest to academics working in areas in which non-systematic reviews remain the predominant synthesis approach. However, there are certain potential limitations we should address. Our review was limited to seven European languages and seven major databases plus GS and DOAJ, so may not have achieved 100% coverage of global literature. Also, the speed of scientific research and communication remains too slow to offer an effective critique on an ongoing social issue – this work was inspired by an event that was reported in the media in May 2016. In addition, the non-inclusion of grey literature would be considered a limitation by some academics [55, 56]. Nevertheless, Barnish et al. [57] have previously argued against the inclusion of grey literature in certain circumstances, and we believe this is a topic in which it is most appropriate to focus solely on peer-reviewed publications, at least at this stage. Risk of bias assessment could not be conducted in a way that would offer useful comparability due to a wide range of different designs. Therefore, readers should consider their own biases when evaluating this work.

## Conclusion

In this article, we provide the first systematic review of reviews looking at high heels. We also for the first time review the psychosexual literature that provides an invaluable perspective. We also situate our work in the context of recent societal debates.
